# Durability of XBB.1.5 Vaccines against Omicron Subvariants

**DOI:** 10.1056/NEJMc2402779

**Published:** 2024-05-29

**Authors:** Dan-Yu Lin, Yi Du, Yangjianchen Xu, Sai Paritala, Matthew Donahue, Patrick Maloney

**Affiliations:** University of North Carolina Gillings School of Global Public Health, Chapel Hill, NC; Nebraska Department of Health and Human Services, Lincoln, NE; University of North Carolina Gillings School of Global Public Health, Chapel Hill, NC; Nebraska Department of Health and Human Services, Lincoln, NE; Nebraska Department of Health and Human Services, Lincoln, NE; University of Nebraska Medical Center, Omaha, NE

## To the Editor:

On September 11, 2023, the updated Moderna and Pfizer-BioNTech mRNA COVID-19 vaccines containing the SARS-CoV-2 omicron subvariant XBB.1.5 were authorized by the U.S. Food and Drug Administration (FDA) for all doses administered to individuals 6 months of age and older.^1^ On October 3, 2023, the updated Novavax adjuvanted COVID-19 vaccine containing the spike protein from the XBB.1.5 subvariant was authorized by the FDA for use in individuals 12 years of age and older.^2^ Here, we report clinical data on the durability of protection conferred by these updated vaccines against circulating omicron subvariants over a 5-month period.

We collected individual-level data on the uptake of the three XBB.1.5 vaccines and the incidence of COVID-19 between September 11, 2023 and February 21, 2024 in a cohort of ~1.8 million persons by linking records from the Nebraska Electronic Disease Surveillance System and the Nebraska State Immunization Information System (NSIIS) (Supplemental Methods). During this period, the dominant circulating variants changed from EG.5 and XBB.1.16 to HV.1 and then to JN.1, and the proportion of XBB.1.5 decreased from 10% to <1%. In the cohort, 218,250 persons (11.9%) received XBB.1.5 vaccines (61.1% Pfizer-BioNTech, 38.6% Moderna) (Figure S1); 21,988 SARS-CoV-2 infections, 1,364 COVID-19-related hospitalizations, and 237 COVID-19-related deaths were reported (Table S1).

We considered four clinical endpoints: infection, hospitalization, hospitalization or death (whichever occurred first), and death. We fit a Cox regression model to each event time in which the hazard ratio for the updated vaccine depends on the time elapsed since vaccination (Supplemental Methods). To reduce confounding bias caused by changing infection rates over time, we compared the risks of disease between recipients and non-recipients of the XBB.1.5 vaccines on the same date. To further reduce confounding bias, we included time since previous vaccination, time since previous infection, and demographic factors (sex, age, race, ethnicity, and socioeconomic status) as covariates. We measured vaccine effectiveness by one minus the hazard ratio.

**Figure 1.**
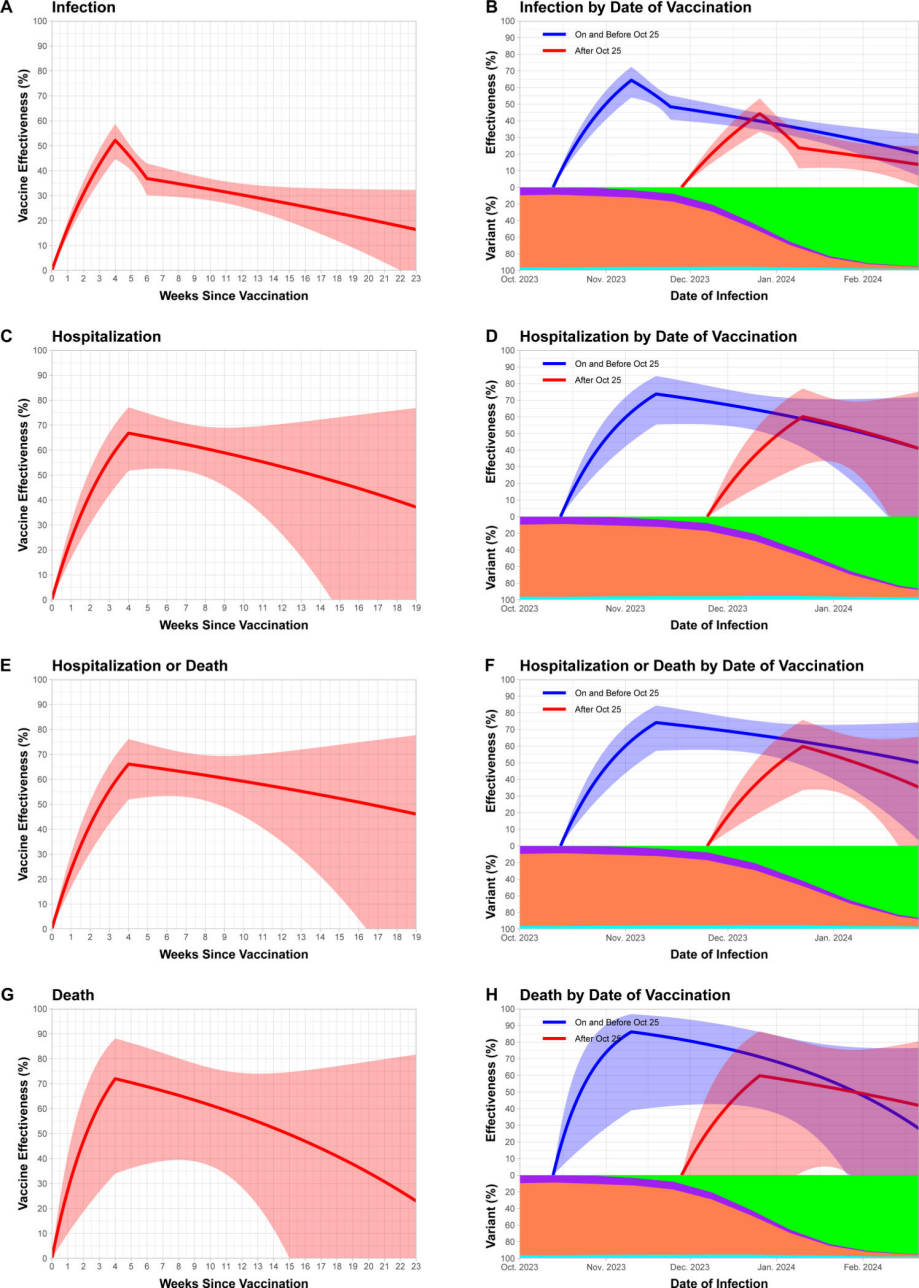
Effectiveness of the XBB.1.5 vaccines against omicron subvariants as a function of time since vaccination. The first, second, third, and fourth rows pertain to the endpoints of infection, hospitalization, hospitalization or death, and death, respectively. The left column pertains to the analysis of all vaccine doses, and the right column pertains to the stratified analysis by vaccination cohort (i.e., receipt date of the XBB.1.5 vaccine). The solid curves show the estimates of vaccine effectiveness. The shaded bands indicate 95% confidence intervals. In (B), (D), (F), and (H), each curve starts at the median receipt date of the XBB.1.5 vaccine for persons in that cohort, and the proportions of XBB.1.5, other XBB, JN.1, and other subvariants are indicated by the purple, coral, green, and cyan areas, respectively.

The estimation results are shown in the left column of Figure 1 and in Table S2. Effectiveness against infection reached a level of 52.2% (95% confidence interval [CI], 44.6 to 58.7) after 4 weeks and decreased to 32.6% (95% CI, 28.1 to 36.8) after 10 weeks, and to 20.4% (95% CI, 6.2 to 32.5) after 20 weeks. Effectiveness against hospitalization reached a level of 66.8% (95% CI, 51.7 to 77.1) after 4 weeks and decreased to 57.1% (95% CI, 40.4 to 69.2) after 10 weeks. Effectiveness against death was higher, but with substantial uncertainty due to the small number of events. Additional analyses showed that the XBB.1.5 vaccines were effective across age groups and in persons who had not been previously infected or previously vaccinated (Figure S2).

We also analyzed the data separately for two vaccination cohorts: receiving the XBB.1.5 vaccines on and before versus after October 25, 2023, with approximately the same number of XBB.1.5 vaccine recipients per cohort. The results are shown in the right column of Figure 1 and in Tables S3 and S4. Vaccine effectiveness was lower in the second cohort than in the first cohort, indicating that the XBB.1.5 vaccines were less protective against JN.1 than against XBB sub-lineages.

This study covered mostly symptomatic SARS-CoV-2 infections and did not include at-home test results. We examined all hospital discharge data from member hospitals of Nebraska Hospital Association, and we examined all death certificates in the state of Nebraska to identify COVID-19-related deaths. Although reporting of vaccine administration became optional after the expiration of the Federal COVID-19 Public Health Emergency Declaration, the number of vaccine providers who reported XBB.1.5 vaccination data to the NSIIS was similar to that of the time period when reporting of vaccine doses was mandatory (Supplemental Methods).

Our analysis was limited by confounding bias. We reduced this bias by adjusting for measured baseline risk factors, and we avoided confounding due to time trends by comparing disease incidence between recipients and non-recipients of the XBB.1.5 vaccines on the same date. Sensitivity analyses showed that our statistical adjustment for confounding was successful and our results were robust to modelling choices (Supplemental Appendix).

The statistical analysis plan pre-specified a piecewise linear function for the log hazard ratio (Supplemental Methods). The resulting curve for vaccine effectiveness against infection varied abruptly at changepoints (Figure 1A). A smoother representation is shown in Figure S4A. There was potential for overfitting, so the curves should not be overinterpreted. Specifically, effectiveness likely peaked around 4 weeks, but the data were not dense enough to precisely locate the peak.

In conclusion, the XBB.1.5 vaccines were effective against omicron subvariants, although less so against JN.1. The effectiveness was greater against hospitalization and death than against infection, and it waned moderately from its peak over time. The ramping and waning patterns were broadly similar to those of the bivalent boosters against BQ.1–BQ.1.1 and XBB–XBB.1.5.^3^ It would be worthwhile to develop and deploy new vaccines targeting JN.1 or future strains.

## Supplementary Material

Supplement

## References

[1] Food and Drug Administration. FDA Takes Action on Updated mRNA COVID-19 Vaccines to Better Protect Against Currently Circulating Variants. Press release, September 11, 2023 (https://www.fda.gov/news-events/press-announcements/fda-takes-action-updated-mrna-covid-19-vaccines-better-protect-against-currently-circulating),

[2] Food and Drug Administration. FDA Authorizes Updated Novavax COVID-19 Vaccine Formulated to Better Protect Against Currently Circulating Variants. Pres release, October 03, 2023 (https://www.fda.gov/news-events/press-announcements/fda-authorizes-updated-novavax-covid-19-vaccine-formulated-better-protect-against-currently).

[3] Lin DY, Xu Y, Gu Y, Durability of bivalent boosters against new omicron subvariants. N Engl J Med, 2023; 388:1818–1820. 37043647 10.1056/NEJMc2302462 PMC10120009

