# Supplementary material for: Durability of XBB.1.5 Vaccines against Omicron Subvariants
Source: N Engl J Med. Author manuscript; Available in PMC 2024 Jun 25. (PMC11197991; doi:10.1056/NEJMc2402779)
Supplement: Supplement [file NIHMS1991980-supplement-Supplement.pdf]

Table of Contents

Supplemental Methods.. 1

Sensitivity Analyses ……………………………………………………………………………………....................... 3

References…….................... 4

Table S1. Demographic Characteristics of the Nebraska Population with Regard to Vaccine Uptake and Clinical Outcomes between September 11, 2023 and February 21, 2024 5

Table S2. Estimates (95% CI) for the Effectiveness of XBB.1.5 Vaccines against Infection, Hospitalization, and Death between September 11, 2023 and February 21, 2024 ……………. 6

Table S3. Estimates (95% CI) for the Effectiveness of XBB.1.5 Vaccines against Infection, Hospitalization, and Death for the Cohort Vaccinated between September 11 and October 25, 2023 ……..…… 7

Table S4. Estimates (95% CI) for the Effectiveness of XBB.1.5 Vaccines against Infection, Hospitalization, and Death for the Cohort Vaccinated between October 26, 2023 and February 21, 2024 ………..…… 8

Figure S1. Numbers of the Updated Vaccine Doses Administered over Time ……………………… 9

Figure S2. Effectiveness of XBB.1.5 Vaccines as a Function of Time Since Vaccination, for Subgroups Defined by Age or by Previous Immunity Status …………………………………..……… 10

Figure S3. Effectiveness of the XBB.1.5 Vaccines Against Omicron Infection as a Function of Time since Vaccination, With versus Without the Constraint of Zero Effectiveness at Week 0 …… 11

Figure S4. Effectiveness of the XBB.1.5 Vaccines Against Omicron Subvariants as a Function of Time since Vaccination under a Quadratic Spline Model.…………………………………………………… 12

Figure S5. Effectiveness of the XBB.1.5 Vaccines Against Omicron Subvariants as a Function of Time since Vaccination, Using the American Community Survey Data ………………………………. 13

Supplemental Methods

Data Sources

Nebraska Electronic Disease Surveillance System

The Nebraska Electronic Disease Surveillance System (NEDSS) is a statewide, web-based infectious disease surveillance and case management system used by the Nebraska Department of Health and Human Services and 19 local health departments covering 93 counties. The NEDSS received COVID-19 test results, including polymerase-chain-reaction and antigen tests, from facilities participating in electronic laboratory reporting across Nebraska. During the study period, individuals who were tested for COVID-19 typically presented with symptoms consistent with COVID-19. The records meeting the Council of State and Territorial Epidemiologists' case definition of COVID-19 were identified. Positive tests within 90 days of initial diagnosis were considered the same infection. At-home tests were generally not included. COVID-19-related hospitalizations were identified through hospital discharge data from member hospitals of the Nebraska Hospital Association. All the hospitalizations listing ICD-10-CM diagnosis code "U07.1" in the patients' discharge billing records either in their primary diagnosis column or another diagnosis column were included. We did not consider comorbidities when defining COVID-19-related hospitalizations. We extracted COVID-19-related deaths from death certificates, with COVID as either the underlying or multiple cause of death. Both COVID-19-related hospitalizations and deaths were matched to the electronic laboratory reporting records by using the data linkage process described below.

Nebraska State Immunization Information System

The Nebraska State Immunization Information System (NESIIS) is a statewide, web-based immunization information system that secures COVID-19 vaccine data from public clinics, private provider offices, local health departments, and hospitals. All Nebraska residents 6 months of age and older are eligible to receive the updated COVID-19 vaccines. The NESIIS keeps accurate, up-to-date COVID-19 vaccination records, including types of vaccine (monovalent, bivalent, updated XBB.1.5), dates of vaccine administration, vaccine manufacturers (Moderna, Pfizer-BioNTech, Novavax), number of doses, and vaccine recipient information.

Reporting of vaccine doses became optional after the end of the Federal COVID-19 Public Health Emergency Declaration on May 11, 2023. However, vaccine providers who participate in the Vaccines for Children or Bridge Access programs and pharmacists are required to report all doses administered. Indeed, the number of providers who reported vaccination data to the NSIIS after September 11, 2023 was similar to that before May 11, 2023.

Data Linkage

Laboratory and vaccination records were probabilistically matched by first name, last name, middle name, date of birth, sex, and residential zip code. Match*Pro v2.3 software was used to link the data. The linkage was based on the Fellegi and Sunter model, under which a probabilistic score was used to determine the quality of the match. Records that demonstrated a high total match score, typically above 29.0, were considered to belong to the same individual. The linked records were also reviewed manually.

Analysis Dataset

The analysis dataset was prepared by merging data on COVID-19 laboratory testing results and COVID-19 vaccination records from January 26, 2023 to February 21, 2024, for Nebraska residents with records of vaccination or COVID-19 testing. COVID-19-related hospitalizations that occurred between September 11, 2023 and January 26, 2024 and COVID-19-related deaths that occurred between September 11, 2023 and February 21, 2024 were also included. Demographic data were obtained from the NEDSS and NESIIS and included in the linked dataset. The 2020 vintage bridged-race postcensal population estimates from the National Center for Health Statistics were used to append the vintage postcensal population records on age, sex, race, ethnicity, county of residence, and residential zip code for persons who were not in the NESIIS or NEDSS database (i.e., persons with no records of COVID-19 vaccination or positive COVID-19 diagnosis).

We used the proportions of variants provided by the U.S. Centers for Disease Control and Prevention's national genomic surveillance system: https://covid.cdc.gov/covid-data-tracker/#variant-proportions.

Statistical Analysis Plan

We first fit a Cox regression model in which the hazard ratio of SARS-CoV-2 infection for the updated vaccine depends on the time elapsed since vaccination.[1−3] To reduce confounding bias, we include the following baseline characteristics as covariates: time since previous vaccination (as of September 11, 2023), which was set to 9 months if it was greater than 9 months or if the person was not previously vaccinated; time since previous infection (as of September 11, 2023), which was set to 9 months if it was greater than 9 months or if the person was not previously infected; and the demographic factors of sex, age group, race and ethnicity, and socioeconomic status. We create a socioeconomic index by zip code according to median income, fraction of vacant housing, fraction of poverty, fraction of persons without health insurance, fraction of persons with a high school degree or higher, and fraction of persons with assisted income, with a higher index indicating worse socioeconomic standing.[4] We then dichotomize this index at the sample median to create the "low" versus "high" categories. In addition, we create a race and ethnicity variable by contrasting non-Hispanic white with all others.

Let S denote the time when the person receives the updated vaccine, and T denote the time when the person tests positive for SARS-CoV-2 infection. Both times are measured in days from the start of the study (i.e., September 11, 2023). In addition, let X denote baseline risk factors (i.e., age, sex, race/ethnicity, socioeconomic status, time of previous infection, and time of previous vaccination). We specify that the hazard function of T is related to S and X through the Cox regression model

$$\lambda(t|S, X) = \lambda_0(t)e^{\beta^{\mathrm{T}} X + I(S<t)\eta(t-S)}, \tag{1}$$

where $I(\cdot)$ is the indicator function, $\lambda_0(\cdot)$ is an arbitrary baseline hazard function, β is a set of log hazard ratios representing the effects of baseline risk factors, and $\eta(\cdot)$ is a log hazard ratio function characterizing the time-varying effect of the updated vaccine.[1−3] Because the time to SARS-CoV-2 infection for each person is measured from a common origin, namely September 11, 2023, the risks of infection for the recipients and non-recipients of the updated vaccines

are compared on the same calendar date, such that confounding bias caused by time-varying infection rates is avoided. The receipt of the updated vaccine is a time-varying exposure variable: a subject moves from the "unexposed" group to the "exposed" group when they receives the updated vaccine. The effect of the updated vaccine on the risk of SARS-CoV-2 infection depends on the time elapsed since vaccination.

We approximate the time-varying log hazard ratio $\eta(\cdot)$ in model (1) by a continuous B-spline function with degree one (i.e., continuous piece-wise linear function)[1−3]

$$\gamma_0 + \gamma_1 t + \gamma_2(t - t_1)_+ + \gamma_3(t - t_2)_+ \cdots + \gamma_{m+1}(t - t_m)_+, \tag{2}$$

where $t_+ = t$ if $t > 0$ and 0 otherwise, $t_1, t_2, \ldots, t_m$ are the m pre-specified knots or change-points, and $\gamma_0, \gamma_1, \ldots, \gamma_{m+1}$ are the unknown parameters pertaining to the intercept and the slope of each piece. We omit the intercept γ_0 from (2) since no vaccine takes immediate effect. The approximation by (2) becomes more accurate as the number of change-points increases. However, the number of parameters to be estimated increases with the number of change-points. With the limited number of vaccine recipients and the limited number of infections, we can obtain stable estimates for only a small number of parameters by choosing a small number of change-points. For this study, we place potential change-points at 2 weeks, 4 weeks, and 6 weeks (i.e., 14 days, 28 days, and 42 days), in order to ascertain the ramping-up and waning effects of the vaccine with sufficient precision and stability.

We define the time-varying vaccine effectiveness as one minus the hazard ratio function,[5] i.e., $\text{VE}(t) = 1 - e^{\eta(t)}$. We use maximum partial likelihood to estimate the hazard ratio and the vaccine effectiveness and construct 95% confidence intervals (CIs).[1] We may omit a change-point to reduce variability and improve stability if the estimated VE curve is relatively smooth at that point.

We fit similar Cox regression models to three additional endpoints: time to hospitalization, time to hospitalization or death, whichever occurred first, and time to death. Because of the smaller numbers of events, we place only a single change-point at 4 weeks for the three hazard ratio functions. When analyzing the hospitalization endpoint, we censor time to hospitalization at time of death, such that the Cox regression analysis pertains to a cause-specific hazard function, rather than the usual hazard function.[6]

In the main analysis, we estimate vaccine effectiveness for the three updated vaccines combined. In order to determine whether effectiveness depends on the subvariant, we also perform the analysis separately for two vaccination cohorts with approximately the same number of XBB.1.5 vaccine recipients per period. For the endpoints of time to infection and time to hospitalization or death, we perform analyses for subgroups defined by XBB.1.5 vaccine manufacturer (Moderna, Pfizer-BioNTech), by age ($\geq$ 65 vs <65 years of age), and by previous immunity status. The last variable compares persons who had been vaccinated or infected (or both) within the past 9 months of September 11, 2023 with those who had not been vaccinated or infected within the past 9 months of September 11, 2023. We choose the cut-off of 9 months because the immunity conferred by vaccination or natural infection becomes negligible after 9 months.

Sensitivity Analyses

Figure S3 (panel A) shows the estimated VE curve with change-points at 2, 4, and 6 weeks. Because the VE curve changes gradually at 2 weeks and the VE estimates were similar with

versus without the change-point at 2 weeks, we removed the change-point at 2 weeks in our main analysis, in order to reduce variability and increase stability.

We imposed the constraint that VE(0) = 0 since vaccine has no biological effect on infection at the time of injection. In an observational study, however, VE(0) may not be equal to 0 because of selection bias. Thus, we performed a sensitivity analysis without the constraint of VE(0) = 0, and VE(0) was estimated at –8.1% (95% CI, –36.4 to 14.3) (Figure S3 B). The fact that the point estimate was a small negative number suggests that persons who were at high risk of infection (e.g., immunocompromised persons) were slightly more likely to receive the XBB.1.5 vaccines. However, the confidence interval includes 0. Thus, our statistical adjustment for confounding was successful. We imposed the constraint of VE(0) = 0 in our main analysis in order to reduce variability and increase stability.

Figure S4 shows the estimated VE curves under a quadratic spline model placing a single changepoint at 4 weeks, with a quadratic function before 4 weeks and a linear function after 4 weeks and under the constraint of equal derivatives between the two functions at the changepoint. Compared with the piecewise linear model, this quadratic spline model yielded smoother VE curves, with slightly lower peaks and more gradual rises and drops around the peaks. The basic conclusions are the same between the two models.

We used the 2020 vintage bridged-race postcensal population data from the National Center for Health Statistics to create dummy records on age, sex, race, ethnicity, county of residence, and residential zip code for those with no records of COVID-19 vaccination or positive COVID-19 diagnosis. To explore the robustness of our VE estimates to perturbations in the population counts, we redid the analysis by using the 2022 American Community Survey Data instead. The results barely changed (Figure S5). We decided to use the vintage bridged-race postcensal population data because it has more accurate information on race/ethnicity and age.

References

1. Lin DY, Gu Y, Wheeler B, et al. Effectiveness of Covid-19 vaccines over a 9-month period in North Carolina. N Engl J Med 2022; 386: 933–941.

2. Lin DY, Gu Y, Xu Y, et al. Association of primary and booster vaccination and prior infection with SARS-CoV-2 infection and severe COVID-19 outcomes. JAMA 2022; 328: 1415-1426.

3. Lin DY, Xu Y, Gu Y, et al. Durability of bivalent boosters against omicron subvariants. N Engl J Med 2023; 388: 1818-1820.

4. Brokamp C, Beck AF, Goyal NK, et al. Material community deprivation and hospital utilization during the first year of life: an urban population-based cohort study. Ann Epidemiol 2019; 30:37–43.

5. Durham LK, Longini Jr IM, Halloran ME, et al. Estimation of vaccine efficacy in the presence of waning: application to cholera vaccines. Am J Epid 1998; 147:948-959.

6. Kalbfleisch JD, Prentice RL. The Statistical Analysis of Failure Time Data. John Wiley & Sons, 2011.

Table S1. Demographic Characteristics of the Nebraska Population with Regard to Vaccine Uptake and Clinical Outcomes between September 11, 2023 and February 21, 2024.

Characteristic	Overall	XBB.1.5 Vaccines			Clinical Outcomes		
		Moderna	Pfizer-BioNTech	Novavax	Infection	Hospitalization	Death
	No. (column %)		No. (row %)			No. (row %)	
All persons	1,830,088 (100)	84,307 (4.61)	133,403 (7.29)	540 (0.030)	21,988 (1.20)	1,364 (0.07)	237 (0.013)
Age							
0–11	221,553 (12)	6,488 (2.93)	3,095 (1.40)	1 (0.000)	2,491 (1.12)	23 (0.01)	0 (0)
12–64	1,218,678 (67)	34,021 (2.79)	68,916 (5.65)	383 (0.031)	12,214 (1.00)	356 (0.03)	23 (0.000)
≥65	389,857 (21)	43,798 (11.23)	61,392 (15.75)	156 (0.040)	7,283 (1.87)	985 (0.25)	214 (0.055)
Sex							
Male	896,450 (49)	37,457 (4.18)	57,936 (6.46)	229 (0.026)	8,538 (0.95)	670 (0.08)	105 (0.012)
Female	933,638 (51)	46,850 (5.02)	75,467 (8.08)	311 (0.033)	13,450 (1.44)	694 (0.07)	132 (0.014)
Race and ethnicity							
Non-Hispanic white	1,484,066 (81)	74,138 (5.00)	113,824 (7.67)	500 (0.034)	17,843 (1.20)	1,221 (0.08)	221 (0.015)
All others	346,022 (19)	10,169 (2.94)	19,579 (5.66)	40 (0.012)	4,145 (1.20)	143 (0.04)	16 (0.005)
Socioeconomic status							
Low	998,249 (55)	44,445 (4.45)	75,780 (7.59)	226 (0.023)	11,650 (1.17)	704 (0.07)	123 (0.012)
High	831,839 (45)	39,862 (4.79)	57,623 (6.93)	314 (0.038)	10,338 (1.24)	660 (0.08)	114 (0.014)
Previous vaccination							
<9 months	64,462 (4)	8,845 (13.72)	14,675 (22.77)	48 (0.074)	1,008 (1.56)	65 (0.10)	10 (0.016)
≥9 months[a]	1,765,626 (96)	75,462 (4.27)	118,728 (6.72)	492 (0.028)	20,980 (1.19)	1,299 (0.07)	227 (0.013)
Previous infection							
<9 months	19,272 (1)	1,233 (6.40)	1,828 (9.49)	4 (0.021)	100 (0.52)	14 (0.07)	10 (0.052)
≥9 months[b]	1,810,816 (99)	83,074 (4.59)	131,575 (7.27)	536 (0.030)	21,888 (1.21)	1,350 (0.08)	227 (0.013)

a. Including persons who were not previously vaccinated.
b. Including persons who were not previously infected.

Table S2. Estimates (95% CI) for the Effectiveness of XBB.1.5 Vaccines against Infection, Hospitalization, and Death, as a Function of Time Since Vaccination, between September 11, 2023 and February 21, 2024.

Weeks	Infection	Hospitalization	Hospitalization or Death	Death
0	0.0% (0.0, 0.0)	0.0% (0.0, 0.0)	0.0% (0.0, 0.0)	0.0% (0.0, 0.0)
1	16.8% (13.7, 19.8)	24.1% (16.7, 30.9)	23.7% (16.7, 30.1)	27.2% (9.9, 41.3)
2	30.8% (25.6, 35.7)	42.4% (30.5, 52.2)	41.8% (30.7, 51.1)	47.1% (18.8, 65.5)
3	42.5% (35.8, 48.5)	56.3% (42.1, 66.9)	55.6% (42.3, 65.8)	61.5% (26.8, 79.7)
4	52.2% (44.6, 58.7)	66.8% (51.7, 77.1)	66.1% (51.9, 76.1)	72.0% (34.0, 88.1)
5	45.0% (40.2, 49.5)	65.3% (52.5, 74.7)	65.1% (52.9, 74.1)	70.5% (36.2, 86.3)
6	36.9% (30.2, 42.9)	63.8% (52.6, 72.4)	64.0% (53.3, 72.2)	68.8% (37.9, 84.4)
7	35.8% (29.9, 41.3)	62.3% (51.7, 70.6)	62.8% (53.0, 70.6)	67.1% (39.0, 82.3)
8	34.7% (29.5, 39.6)	60.6% (49.4, 69.4)	61.7% (51.5, 69.7)	65.3% (39.5, 80.1)
9	33.7% (28.9, 38.1)	58.9% (45.6, 69.0)	60.4% (48.9, 69.4)	63.4% (38.9, 78.1)
10	32.6% (28.1, 36.8)	57.1% (40.4, 69.2)	59.2% (45.2, 69.6)	61.4% (37.1, 76.4)
11	31.4% (27.0, 35.6)	55.3% (34.0, 69.7)	57.9% (40.5, 70.2)	59.3% (33.7, 75.1)
12	30.3% (25.5, 34.8)	53.3% (26.3, 70.5)	56.6% (35.0, 71.0)	57.1% (28.5, 74.3)
13	29.1% (23.8, 34.1)	51.3% (17.3, 71.3)	55.2% (28.6, 71.9)	54.8% (21.2, 74.0)
14	28.0% (21.8, 33.7)	49.2% (7.0, 72.2)	53.8% (21.3, 72.9)	52.3% (11.8, 74.2)
15	26.8% (19.5, 33.3)	47.0% (-4.8, 73.2)	52.4% (13.1, 73.9)	49.7% (0.1, 74.7)
16	25.5% (17.1, 33.1)	44.7% (-18.3, 74.1)	50.9% (4.0, 74.8)	46.9% (-14.2, 75.3)
17	24.3% (14.6, 32.9)	42.3% (-33.6, 75.1)	49.3% (-6.2, 75.8)	44.0% (-31.3, 76.1)
18	23.0% (11.9, 32.7)	39.8% (-51.0, 76.0)	47.7% (-17.6, 76.8)	41.0% (-51.6, 77.0)
19	21.8% (9.1, 32.6)	37.1% (-70.8, 76.9)	46.1% (-30.3, 77.7)	37.7% (-75.7, 77.9)
20	20.4% (6.2, 32.5)			
21	19.1% (3.2, 32.4)			
22	17.8% (0.1, 32.4)			
23	16.4% (-3.2, 32.3)			

Table S3. Estimates (95% CI) for the Effectiveness of XBB.1.5 Vaccines against Infection, Hospitalization, and Death, as a Function of Time Since Vaccination, for the Cohort Vaccinated between September 11 and October 25, 2023.

Weeks	Infection	Hospitalization	Hospitalization or Death	Death
0	0.0% (0.0, 0.0)	0.0% (0.0, 0.0)	0.0% (0.0, 0.0)	0.0% (0.0, 0.0)
1	22.7% (17.6, 27.5)	28.4% (18.3, 37.3)	28.7% (19.1, 37.1)	39.0% (11.6, 57.9)
2	40.3% (32.2, 47.5)	48.7% (33.2, 60.7)	49.1% (34.5, 60.4)	62.8% (21.9, 82.3)
3	53.9% (44.1, 61.9)	63.3% (45.4, 75.3)	63.7% (47.0, 75.1)	77.3% (31.0, 92.6)
4	64.4% (54.0, 72.4)	73.7% (55.4, 84.5)	74.1% (57.1, 84.4)	86.2% (39.0, 96.9)
5	57.1% (50.7, 62.7)	71.7% (55.7, 81.9)	72.5% (57.7, 82.2)	84.6% (40.4, 96.0)
6	48.5% (40.7, 55.2)	69.6% (55.5, 79.2)	70.8% (57.8, 79.8)	82.7% (41.6, 94.9)
7	46.7% (39.6, 52.9)	67.2% (54.6, 76.3)	69.0% (57.4, 77.5)	80.7% (42.4, 93.5)
8	44.8% (38.5, 50.5)	64.7% (52.4, 73.8)	67.1% (56.0, 75.4)	78.4% (42.7, 91.8)
9	42.9% (37.1, 48.2)	62.0% (48.5, 72.0)	65.1% (53.3, 73.9)	75.8% (42.5, 89.8)
10	40.9% (35.6, 45.8)	59.1% (42.4, 71.0)	62.9% (49.0, 73.1)	72.9% (41.3, 87.5)
11	38.9% (33.8, 43.6)	56.0% (34.2, 70.6)	60.6% (43.1, 72.8)	69.7% (38.8, 85.0)
12	36.8% (31.6, 41.6)	52.7% (23.7, 70.6)	58.2% (35.7, 72.8)	66.1% (34.4, 82.5)
13	34.6% (29.0, 39.8)	49.0% (10.7, 70.9)	55.6% (26.6, 73.2)	62.1% (27.2, 80.3)
14	32.3% (25.9, 38.2)	45.1% (-5.0, 71.3)	52.9% (15.8, 73.6)	57.6% (16.4, 78.5)
15	30.0% (22.5, 36.7)	40.9% (-23.8, 71.8)	50.0% (3.1, 74.2)	52.6% (0.9, 77.3)
16	27.6% (18.7, 35.4)			46.9% (-20.4, 76.6)
17	25.0% (14.6, 34.2)			40.6% (-49.0, 76.3)
18	22.4% (10.2, 33.1)			33.6% (-86.7, 76.4)

Table S4. Estimates (95% CI) for the Effectiveness of XBB.1.5 Vaccines against Infection, Hospitalization, and Death, as a Function of Time Since Vaccination, for the Cohort Vaccinated between October 26, 2023 and February 21, 2024.

Weeks	Infection	Hospitalization	Hospitalization or Death	Death
0	0.0% (0.0, 0.0)	0.0% (0.0, 0.0)	0.0% (0.0, 0.0)	0.0% (0.0, 0.0)
1	13.6% (9.7, 17.4)	20.5% (8.8, 30.7)	20.4% (9.7, 29.8)	20.4% (-4.6, 39.4)
2	25.4% (18.4, 31.7)	36.9% (16.9, 52.0)	36.6% (18.5, 50.7)	36.6% (-9.5, 63.3)
3	35.5% (26.3, 43.6)	49.8% (24.2, 66.8)	49.5% (26.4, 65.3)	49.6% (-14.5, 77.8)
4	44.3% (33.5, 53.4)	60.1% (30.9, 77.0)	59.8% (33.5, 75.7)	59.8% (-19.8, 86.5)
5	34.8% (27.3, 41.6)	56.8% (33.5, 71.9)	55.6% (33.6, 70.4)	58.0% (-7.7, 83.6)
6	23.8% (11.4, 34.4)	53.1% (28.6, 69.2)	51.1% (29.3, 66.1)	56.0% (0.8, 80.5)
7	22.2% (11.8, 31.4)	49.2% (14.0, 70.0)	46.0% (18.5, 64.3)	54.0% (5.0, 77.7)
8	20.6% (11.6, 28.7)	44.9% (-10.4, 72.5)	40.5% (0.3, 64.4)	51.9% (4.1, 75.9)
9	19.0% (10.6, 26.6)	40.3% (-45.9, 75.6)	34.3% (-25.9, 65.7)	49.7% (-2.9, 75.4)
10	17.4% (8.5, 25.4)			47.3% (-16.8, 76.2)
11	15.7% (5.3, 24.9)			44.9% (-37.9, 78.0)
12	13.9% (1.3, 25.0)			42.3% (-67.4, 80.1)

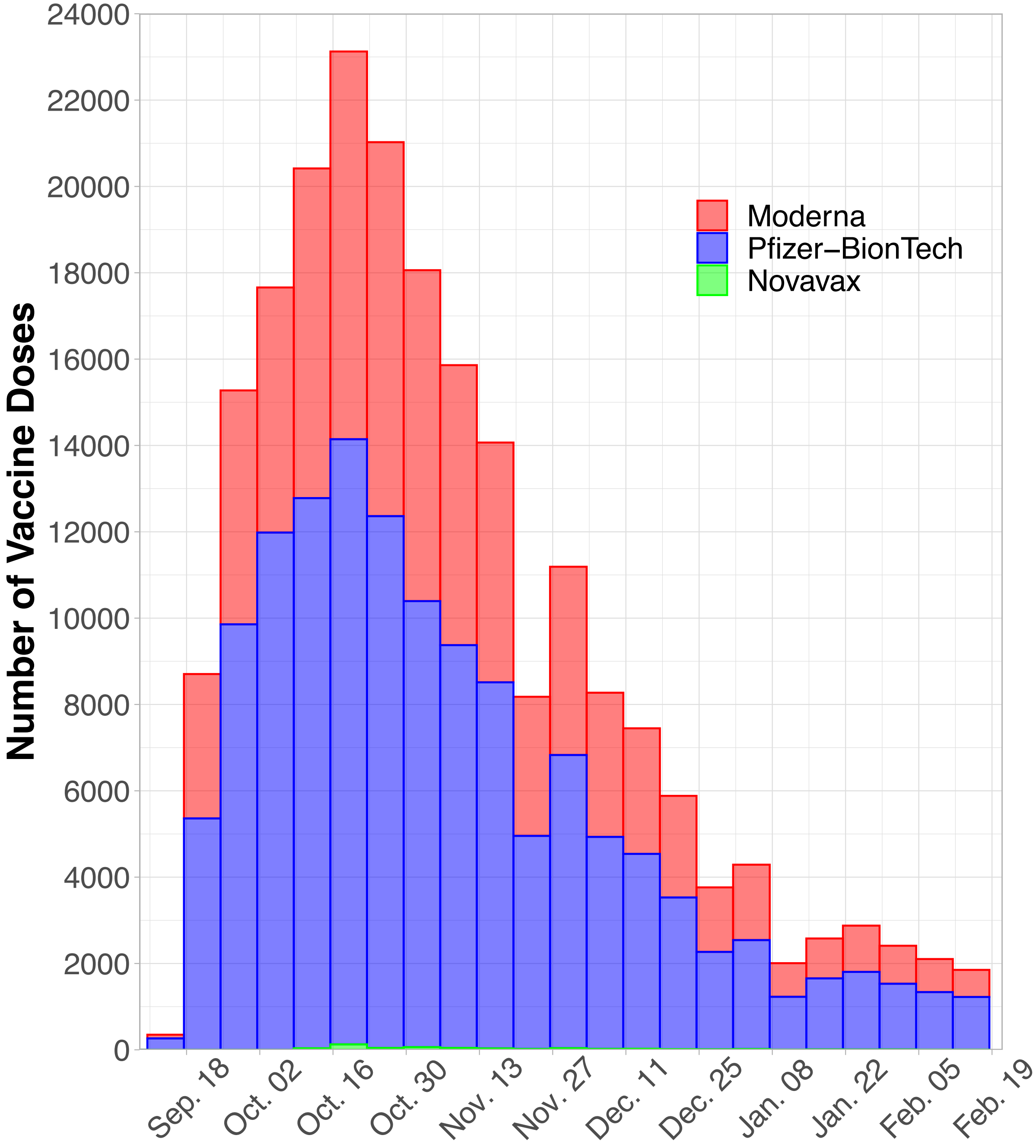

Figure S1. Numbers of the Updated Vaccine Doses Administered over Time.

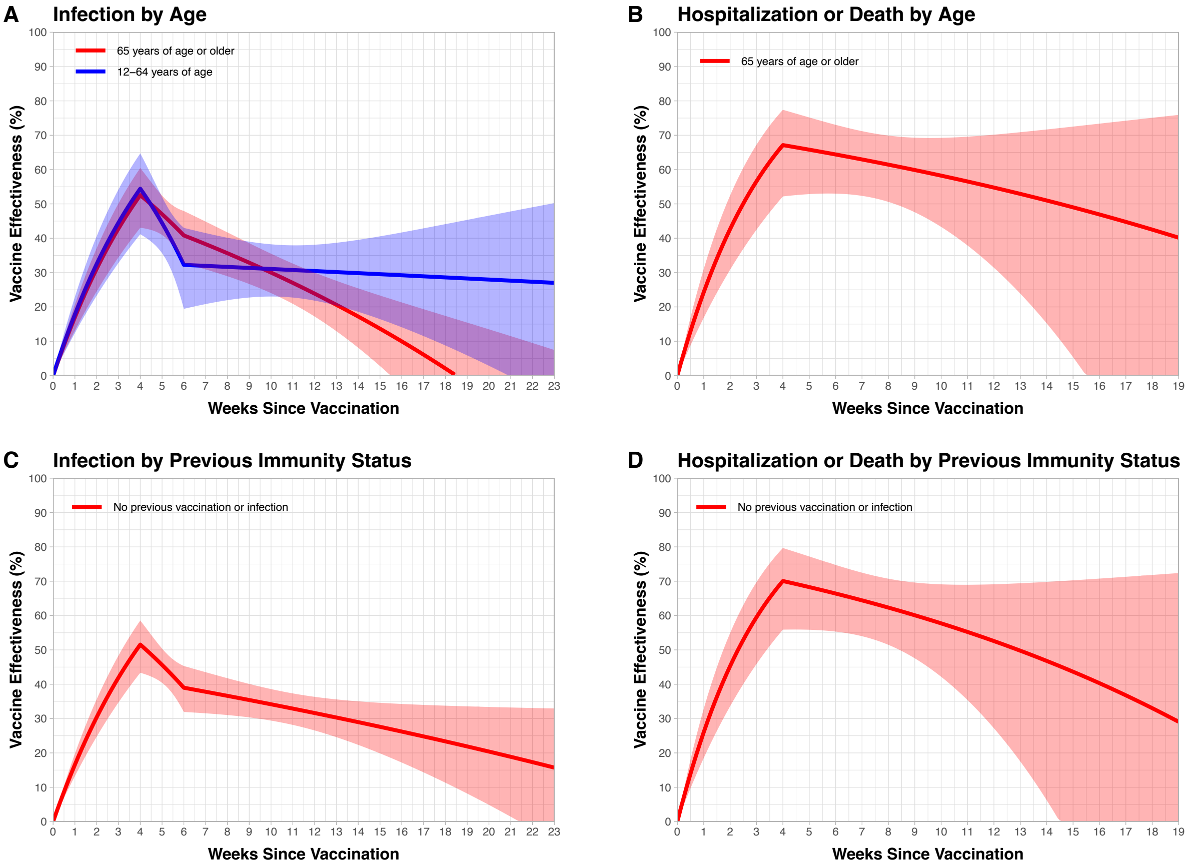

Figure S2. Effectiveness of XBB.1.5 Vaccines as a Function of Time Since Vaccination, for Subgroups Defined by Age or by Previous Immunity Status. The solid curves show the estimates of vaccine effectiveness. The shaded bands indicate 95% confidence intervals. In panel B, the estimate for persons<65 years of age is not shown because of the small number of events. In panels C and D, the estimates for persons with previous vaccination or infection are not shown because of the small sample size.

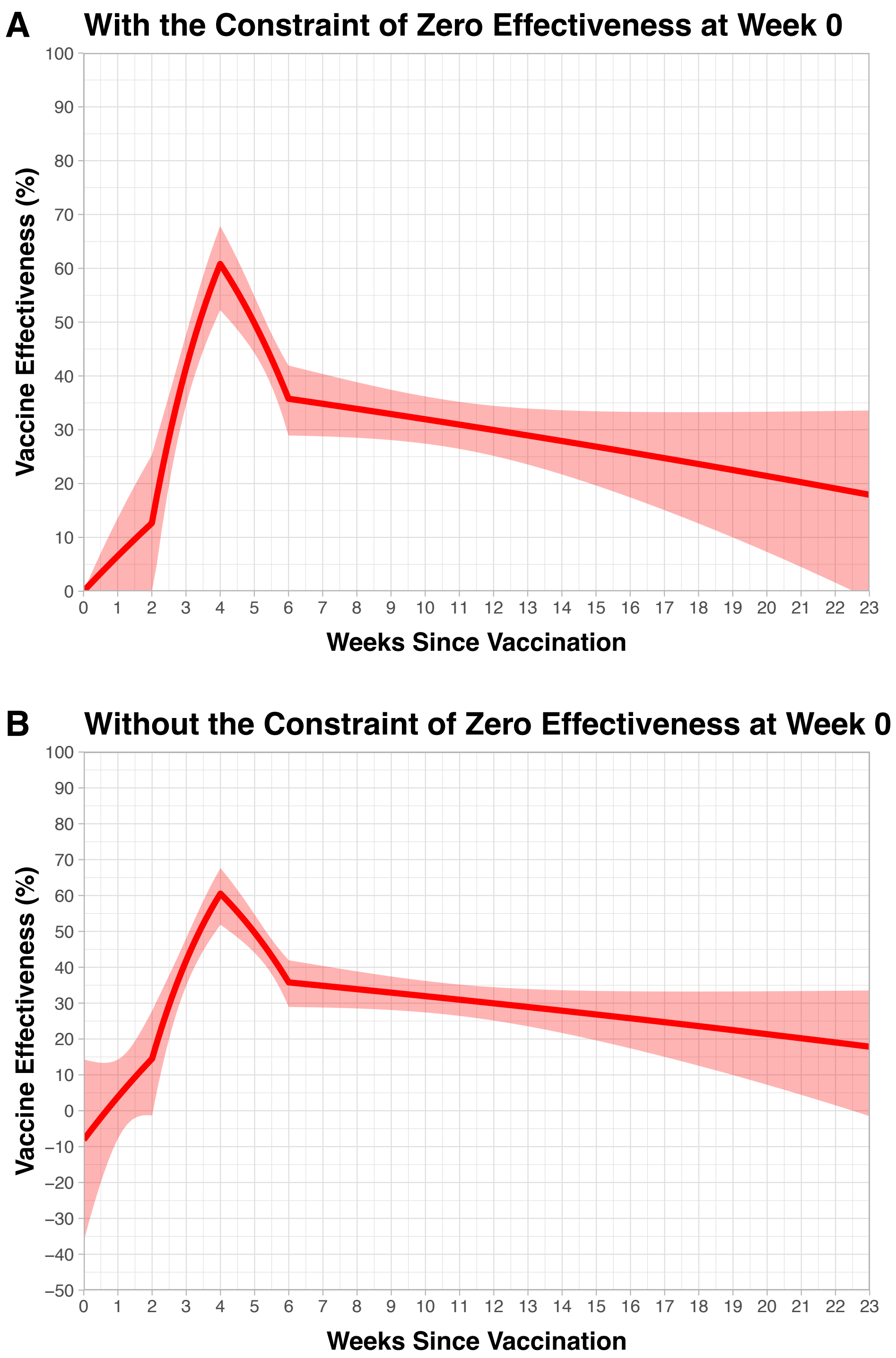

Figure S3. Effectiveness of the XBB.1.5 Vaccines Against Omicron Infection as a Function of Time since Vaccination, With versus Without the Constraint of Zero Effectiveness at Week 0. The solid curves show the estimates of vaccine effectiveness. The shaded bands indicate 95% confidence intervals.

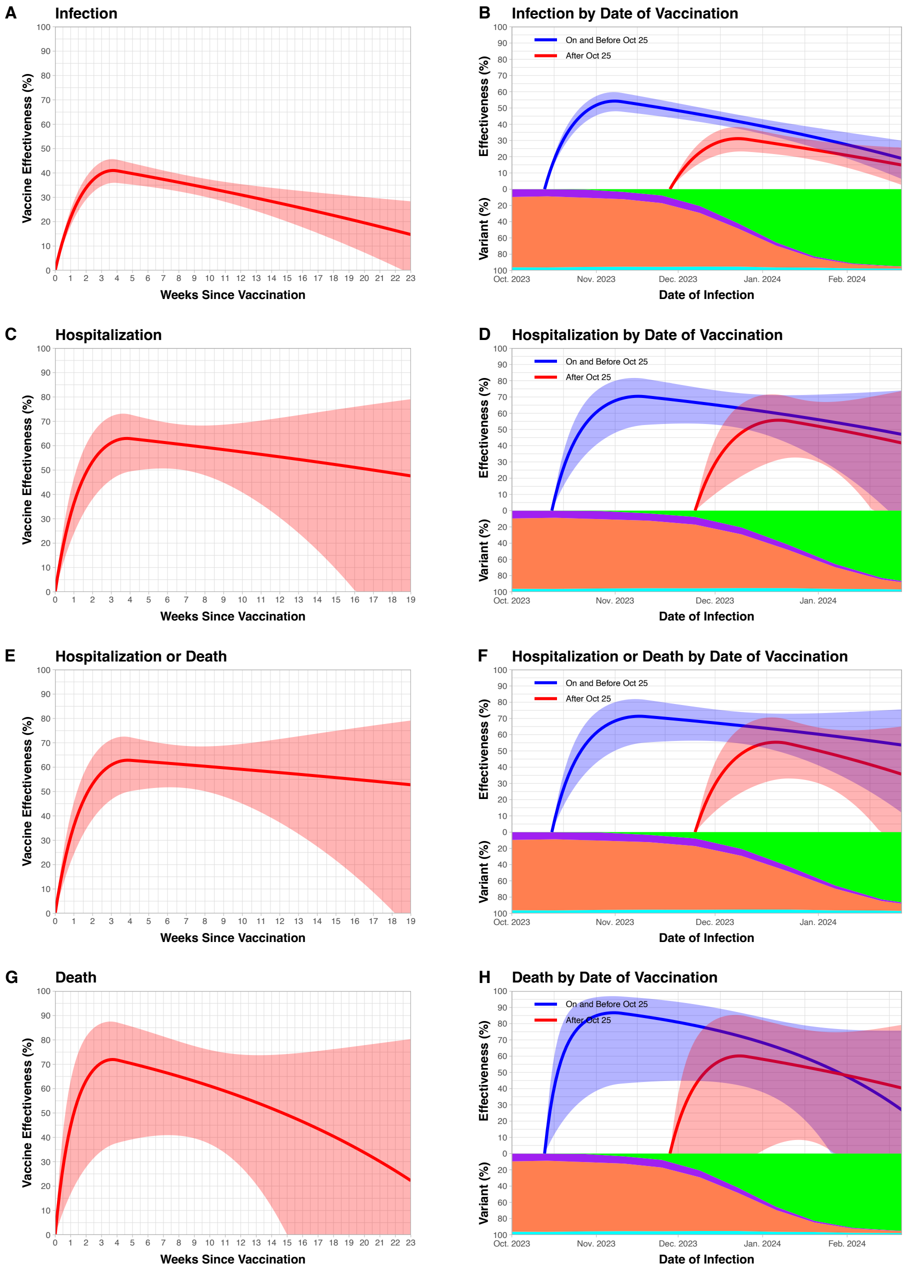

Figure S4. Effectiveness of the XBB.1.5 Vaccines Against Omicron Subvariants as a Function of Time since Vaccination under a Quadratic Spline Model. The first, second, third, and fourth rows pertain to the endpoints of infection, hospitalization, hospitalization or death, and death, respectively. The left column pertains to the analysis of all vaccine doses, and the right column pertains to the stratified analysis by vaccination cohort (i.e., receipt date of the XBB.1.5 vaccine). The solid curves show the estimates of vaccine effectiveness. The shaded bands indicate 95% confidence intervals. In (B), (D), (F), and (H), each curve starts at the median receipt date of the XBB.1.5 vaccine for persons in that cohort, and the proportions of XBB.1.5, other XBB, JN.1, and other subvariants are indicated by the purple, coral, green, and cyan areas, respectively.

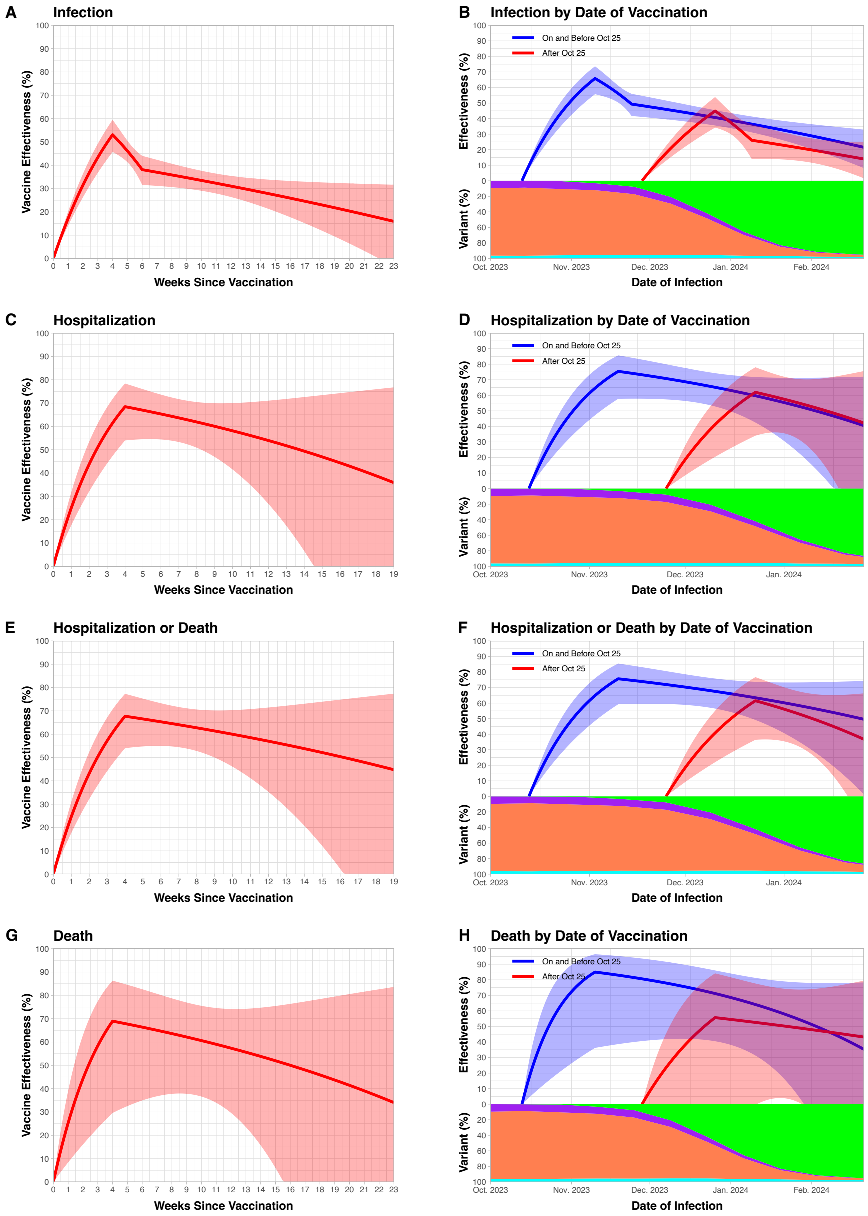

Figure S5. Effectiveness of the XBB.1.5 Vaccines Against Omicron Subvariants as a Function of Time since Vaccination, Using the American Community Survey Data. The first, second, third, and fourth rows pertain to the endpoints of infection, hospitalization, hospitalization or death, and death, respectively. The left column pertains to the analysis of all vaccine doses, and the right column pertains to the stratified analysis by vaccination cohort (i.e., receipt date of the XBB.1.5 vaccine). The solid curves show the estimates of vaccine effectiveness. The shaded bands indicate 95% confidence intervals. In (B), (D), (F), and (H), each curve starts at the median receipt date of the XBB.1.5 vaccine for persons in that cohort, and the proportions of XBB.1.5, other XBB, JN.1, and other subvariants are indicated by the purple, coral, green, and cyan areas, respectively.
